# Supplementary material for: Conditional chemoconnectomics (cCCTomics) as a strategy for efficient and conditional targeting of chemical transmission
Source: eLife. 2024 Apr 30;12:RP91927. doi: 10.7554/eLife.91927 (PMC11060718; doi:10.7554/eLife.91927)
Supplement: Figure 3—source data 3. [file elife-91927-fig3-data3.zip › Figure 3 source data3 part 2.zip!/Figure 3-Source data 3-part2/1053-1-CRISPResso_Report_mpeqg9da.zip!/CRISPResso_on_1053_1/9.Alleles_frequency_table_around_sgRNA_ggagcggaatctacaaaacg.pdf]

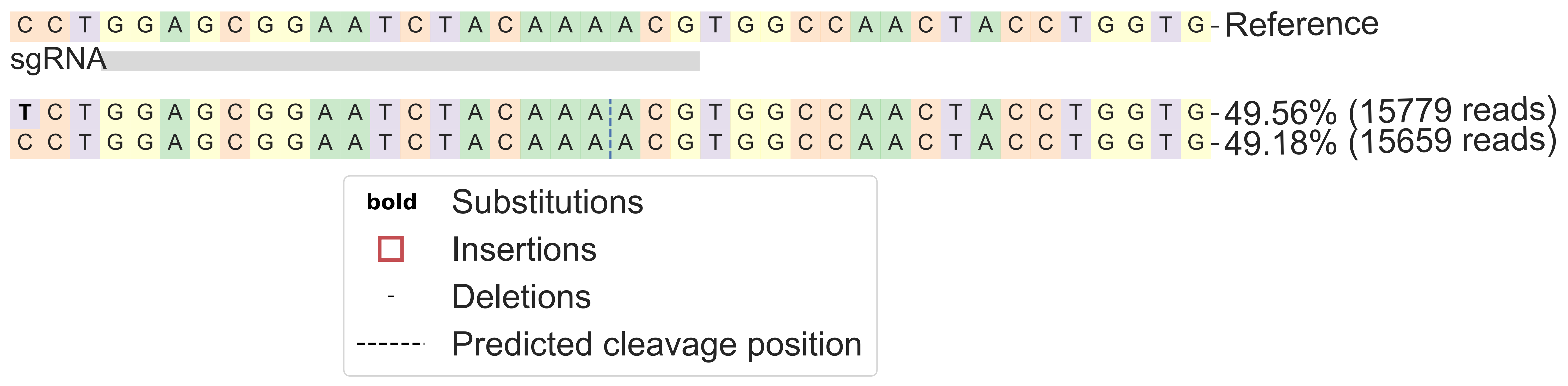
CCTGGAGCGGAATCTACAAAACGTGGCCAACTACCTGGTG - Reference
sgRNA
TCTGGAGCGGAATCTACAAAACGTGGCCAACTACCTGGTG - 49.56% (15779 reads)
CCTGGAGCGGAATCTACAAAACGTGGCCAACTACCTGGTG - 49.18% (15659 reads)
bold Substitutions
Insertions
- Deletions
Predicted cleavage position
